# Ras-transformation reduce FAM20C expression and osteopontin phosphorylation

**DOI:** 10.1042/BSR20194378

**Published:** 2020-09-16

**Authors:** Gitte N. Schytte, Brian Christensen, Ida Bregenov, Esben S. Sørensen

**Affiliations:** 1 Department of Molecular Biology and Genetics, Science Park, Aarhus University, Aarhus, Denmark; 2 Interdisciplinary Nanoscience Center, Aarhus University, Aarhus, Denmark

**Keywords:** FAM20C, mRNA, osteopontin

## Abstract

Family with sequence similarity 20, member C (FAM20C) is the main kinase of secreted phosphoproteins, including the multifunctional protein and cytokine, osteopontin (OPN). The phosphorylation of OPN varies greatly among cell types, tissues and species, and the different phospho-isoforms contribute to the multifunctionality of the protein. Expression of OPN is increased in human malignancies, and less phosphorylated isoforms of the protein have been associated with this phenotype. Here, we compared OPN from *ras*-transformed fibroblasts with that from their non-transformed parental cells, and found that OPN was less phosphorylated after *ras*-transformation. Furthermore, we demonstrated that expression of FAM20C mRNA was reduced five-fold in *ras*-transformed fibroblasts compared with non-transformed fibroblasts. Transfection with FAM20C of the *ras*-transformed fibroblasts restored the FAM20C mRNA expression but the phosphorylation of OPN was not increased proportionally. Likewise, the mRNA level of FAM20C was reduced in the malignant *ras*-transformed mammary cell line MCF10ACA1a compared with its non-transformed parental cell line MCF10A. These results suggest that expression of the FAM20C kinase is reduced after oncogenic *ras*-transformation, which potentially affects the phosphorylation of secreted phosphoproteins.

## Introduction

Family with sequence similarity 20, member C (FAM20C) was recently identified as the long-sought Golgi kinase that phosphorylates secreted proteins [1,2]. FAM20C is ubiquitously expressed in tissues such as the mammary gland, brain, kidney, spleen, liver and mineralized tissues [1,3]. FAM20C has been characterized as an atypical serine/threonine kinase with the canonical recognition motif SXE/pS (phosphoserine); a unique specificity among known kinases [1,4]. FAM20C is the main kinase of secreted proteins and it phosphorylates proteins participating in a wide variety of physiological processes [4]. Several of these processes are impaired by FAM20C loss-of-function mutations. For instance, abrogated FAM20C phosphorylation of fibroblast growth factor 23 inhibits its proteolytic inactivation which results in a pathological hypophosphatemia condition [5]. Furthermore, FAM20C-mediated phosphorylation of oocyte secreted bone morphogenetic protein 15 and growth differentiation factor 9 is essential for regulation of their activity in folliculogenesis and ovulation [6]. Likewise, FAM20C-mediated phosphorylation of von Willebrand factor increased platelet adhesion [7], and lack of FAM20C phosphorylation of histidine-rich calcium binding protein causes ventricular arrhythmia [8].

The multifunctional, integrin-binding and highly phosphorylated protein osteopontin (OPN) is a well-described substrate for FAM20C [1,4]. OPN plays a key role in many physiological processes such as immunolological signaling, biomineralization, tissue remodeling and tumorigenesis [9,10]. Many of these processes are affected by phosphorylation of OPN. In biomineralization assays, highly phosphorylated OPN has been shown to promote hydroxyapatite formation and growth, whereas OPN with fewer phosphorylations inhibits hydroxyapatite formation [11]. The ability of OPN to engage in integrin-mediated cell adhesion is also dependent on the phosphorylation state. Human melanoma MDA-MB-435 cells only adhere weakly to highly phosphorylated OPN, whereas they adhere strongly to less phosphorylated murine *ras-*transformed fibroblast OPN [12]. Likewise, phosphorylation of the N-terminal part of OPN is required for its binding to the β_3_-integrin receptor on macrophages leading to stimulation of interleukin-12 expression [13].

OPN expression is increased in several human cancers [14–16] and OPN participates in proliferation, migration, adhesion, anti-apoptosis and angiogenesis, which are all processes involved in tumorgenesis and metastatis [17,18]. Likewise, OPN expression is increased in transformations with the common proto-oncogene *ras* [19,20]. Mutation of the *ras* gene is observed in approximately 30% of human cancers [21]. The promoter of the OPN gene contains a *ras*-responsive enhancer element [19] which could explain the increased expression of OPN in cancer. Both cancer cells and macrophages have been suggested to contribute to the increased level of OPN in tumors [16]. However, the OPN isoforms secreted from the two cell types appear to be different, and it has been suggested that the cancer-promoting properties of OPN are mainly related to the tumor-derived isoform of OPN [16,22,23].

Several studies have demonstrated that *ras*-transformation results in a less phosphorylated isoform of OPN [12,24]. However, the effect of *ras*-transformation has been deduced from comparisons of OPN from non-transformed cells and *ras*-transformed cells not originating directly from the parental non-transformed cell [12,24]. The degree of OPN phosphorylation varies significantly among cell types [25–28], therefore it is not possible to determine whether the difference in OPN phosphorylation are caused by the *ras*-transformation or whether it is simply due to differences among the analyzed cell types.

In the present study, we compare OPN phosphorylation in *ras*-transformed cells with their non-transformed parental cells. Furthermore, we demonstrate for the first time that the expression of the FAM20C kinase is significantly reduced in *ras*-transformed cells, and that transfection with FAM20C re-establishes the mRNA expression levels, whereas it does not restore the impaired OPN phosphorylation observed in *ras*-transformed cells.

## Materials and methods

### Cell lines and culture conditions

The murine embryonic fibroblast cell line MEF3T3-275 and the *ras*-transformed derivative MEF3T3-275-3-2 [29] (kind gifts from David T. Denhardt, Rutgers University, NJ) were maintained in Dulbecco’s modified Eagle’s medium (DMEM) with Glutamax supplemented with 10% fetal bovine serum and 1% penicillin/streptomycin (all from Gibco). The human fibrocystic mammary epithelial cell line MCF10A [30] and the fully malignant MCF10ACA1a counterpart [31] (kind gifts from Ernst-Martin Füchtbauer, Aarhus University, Denmark) were cultured in a 1:1 mix of DMEM and Ham’s F12 nutrient mixture medium, 5% horse serum and 1% penicillin/streptomycin (all from Gibco). The MCF10ACA1a cell line was further supplemented with 0.5 µg/ml hydrocortisone (Merck), 10 µg/ml insulin (Gibco) and 20 ng/ml recombinant human epidermal growth factor (Gibco).

### Plasmid construction and transfection of cells

FAM20C-pcDNA3.1/myc-His(-)A was generated from human cDNA encoding residues 1–584 of FAM20C (clone ID: 4942737, GE Healthcare). FAM20C cDNA was amplified with the following primers; forward: 5′-ACCCAAGCTGGCTAGATATGAAGATGATGCTGGTGCGCCG-3′ and reverse: 5′-GTTCGGGCCCAAGCTTCCTCGCCGAGGCGGCTCTG-3′ containing a NheI and a HindIII restriction sites, respectively (LGC Biosearch Technologies). Amplified cDNA was recombined into pcDNA3.1/myc-His(-)A (Invitrogen) using the InFusion Cloning Technology (Clontech). Plasmid was propagated in Stellar *Escherichia coli* (Clontech) followed by purification with the PureLink HiPure Plasmid Filter Maxiprep (Invitrogen). MEF3T3-275-3-2 cells were transfected at approximately 70% confluence where the medium was changed to serum-free medium in T25 culture flasks. Four micrograms of FAM20C-pcDNA3.1/myc-His(-)A was reacted with Lipofectamine LTX and Plus reagent (Invitrogen) and supplied to the cells. Conditioned medium was harvested after 48 h.

### SDS/PAGE and Western blot analysis

Conditioned medium was subjected to SDS/PAGE on 16% tris-tricine gels or 10% bis-tris gels (Invitrogen). Separated proteins were transferred to polyvinylidene difluoride membranes and incubated with 1 µg/ml monoclonal mouse anti-human OPN antibodies MAB193p (Maine Biotechnology Services), 2A1 (human epitope ^190^PVA^192^) or 3D9 (human epitope ^283^KFRISHELDSASSEVN^298^) [32] (from David T. Denhardt, Rutgers University, NJ). OPN was detected with alkaline phosphatase (ALP)-conjugated secondary rabbit anti-mouse antibody (1:5000) (Merck). Dephosphorylation was performed by incubating OPN with bovine ALP (Merck) (1 μg OPN: 30 mU ALP) in 50 mM ammonium bicarbonate at 37ºC overnight. The band intensities of phosphorylated and non-phosphorylated OPN were quantified using ImageJ software (National Institutes of Health).

MEF3T3-275 and MEF3T3-275-3-2 cells were washed twice with ice-cold PBS and incubated with radioimmunoprecipitation assay buffer (Merck) for 10 min on ice and centrifuged for 5 min at 9000×***g***. A total of 10 µl cell lysate was separated on 10% bis-tris gels, followed by Western blotting using 1 µg/ml polyclonal rabbit anti-FAM20C (Novus Biologicals) antibodies or a monoclonal mouse β-actin antibody (1:2000) (Merck). A total of 500 ng recombinant human FAM20C (R&D Systems) was loaded as control for the kinase. FAM20C was detected with horseradish peroxidase-conjugated secondary immunoglobulins with enhanced chemiluminescence on an ImageQuant LAS 4000 instrument (GE Healthcare) and β-actin was detected with ALP-conjugated secondary rabbit anti-mouse antibodies (1:5000).

### OPN ELISA

A MaxiSorp immunoassay plate (Thermo Fisher Scientific) was coated overnight at 4°C with the monoclonal MAB193p antibody (5 μg/ml in 0.1 M of sodium carbonate, pH 9.8). The plate was washed extensively with PBS and blocked with 2% ovalbumin (Merck) in PBS for 1 h at 37°C and washed three times with the washing buffer (PBS containing 0.1% Tween 20). Conditioned medium from the MEF3T3-275 and MEF3T3-275-3-2 cells was diluted ten-fold in PBS and incubated with biotinylated 2A1 antibody (2.5 µg/ml) for 1 h at 37°C. Next, the samples were added to the plate and incubated for 1 h at 37°C followed by extensive washing. Captured OPN was detected by incubation with 100 μl of horseradish peroxidase-conjugated streptavidin (diluted 1:10000) for 1 h at 37°C. After the final wash, TMB-one Peroxidase Substrate (Kem-En-Tec Diagnostics) was added and the plate was incubated for 15 min, after which the reaction was quenched by addition of 0.2 M H_2_SO_4_. The plate was read at 450 nm using an ELISA reader (Bio-tek Instruments Inc).

### Quantitative reverse transcription PCR

Total RNA was isolated using the RNeasy Mini Kit (Qiagen) and cDNA was synthesized using the RevertAid H Minus First Strand cDNA Synthesis Kit (Thermo Scientific). Quantitative reverse transcription PCR (qRT-PCRs) were performed with the StepOnePlus Real-Time PCR System (Applied Biosystems). TaqMan Fast Advance Master Mix was applied along with the following predesigned primer- and probe sets; TaqMan Gene Expression Assays (from Applied Biosystems); mouse FAM20C (Mm00504210_m1), mouse OPN (Mm00436767_m1), mouse β-actin (Mm00607939_s1), human FAM20C (Hs00398243_m1), human β-actin (Hs99999903_m1) and human OPN (Hs00959010_m1). mRNA expression levels of FAM20C and OPN were normalized to the endogenous control β-actin and the relative gene expression was presented as 2^**−**ΔΔ*C*_T_^.

### Statistical analysis

Statistical analysis of qRT-PCR data was performed by Welch’s two-tailed unpaired *t* test in GraphPad Prism 6 (GraphPad Software). Calculated differences were considered statistically significant at *P*-values of ≤0.05.

## Results

### FAM20C mRNA expression and OPN phosphorylation in *ras*-transformed fibroblasts

**Figure 1:**
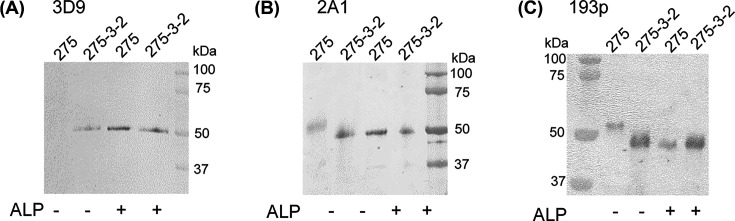
Phosphorylation of OPN in 275 and *ras*-transformed 275-3-2 cells OPN phosphorylation was examined by separating proteins from conditioned medium on a 16% tris-tricine gel by SDS/PAGE followed by Western blotting using the monoclonal OPN antibodies: (**A**) 3D9, (**B**) 2A1 and (**C**) 193p. Dephosphorylation using ALP is indicated. The Western blot is representative of four independent experiments.

To investigate the effect of *ras*-transformation on OPN phosphorylation, OPN from murine embryo fibroblast 275 cells (fOPN) was compared with OPN from the *ras-*transformed derivative 275-3-2 cell (fOPN^ras^). Conditioned medium from the two cell lines was analyzed by Western blotting with the monoclonal OPN antibodies 193p, 2A1 and 3D9. The epitope recognized by 193p and 2A1 contains no phosphorylation sites and they therefore bind OPN irrespective of phosphorylation [32,33]. The epitope recognized by the 3D9 antibody contains several potential phosphorylation sites and its binding to OPN is abrogated when the epitope is phosphorylated [32]. The 3D9 antibody recognized fOPN^ras^ from *ras*-transformed 275-3-2 cells whereas fOPN from 275 cells was not recognized. Upon dephosphorylation with ALP, fOPN was recognized by the 3D9 antibody (Figure 1A). This indicates that fOPN was phosphorylated, whereas fOPN^ras^ was not. This was further substantiated by differences in migration visualized by Western blotting with the 2A1 and 193p antibodies. fOPN migrated at a higher molecular mass compared with fOPN^ras^, and ALP treatment shifted the migration pattern of fOPN to be similar to that of fOPN^ras^ (Figure 1B,C) indicating that fOPN^ras^ was less phosphorylated than fOPN.

**Figure 2:**
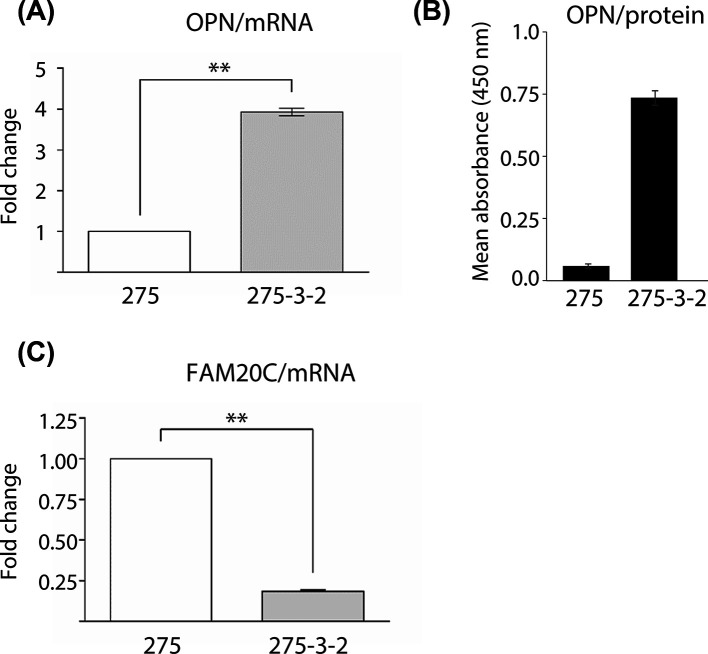
Expression of FAM20C and OPN in 275 and *ras*-transformed 275-3-2 cells (**A**) OPN and (**C**) FAM20C mRNA levels from 275 and *ras*-transformed 275-3-2 cells. FAM20C mRNA levels from 275 and *ras*-transformed 275-3-2 cells. Relative levels were quantified by qRT-PCR analysis. Expression from 275 cells is set as reference and expression from *ras*-transformed 275-3-2 cells is presented as mean ± S.D. (*n*=3). ** indicates *P*<0.0001. (**B**) OPN in cell-conditioned medium was detected by biotinylated 2A1 in maxisorp plates coated with the monoclonal 193p antibody. The data are expressed as mean ± S.D. (*n*=3). The data are representative of three experiments.

To investigate whether the OPN mRNA expression was increased after *ras*-transformation, qRT-PCR analysis was performed on 275 and *ras*-transformed 275-3-2 cells. The mRNA expression of OPN was increased four-fold in *ras*-transformed 275 cells compared with 275 cells (Figure 2A). This correlated with a significant increase in the protein levels of OPN in the medium conditioned by the *ras*-transformed 275-3-2 cells compared with the 275 cells (Figure 2B). In addition, the FAM20C mRNA expression in *ras*-transformed 275-3-2 cells was reduced five-fold compared with the level in the 275 cells (Figure 2C). This could indicate a correlation between a decrease in the mRNA expression of FAM20C and the reduced phosphorylation of OPN in *ras*-transformed 275-3-2 cells.

**Figure 3:**
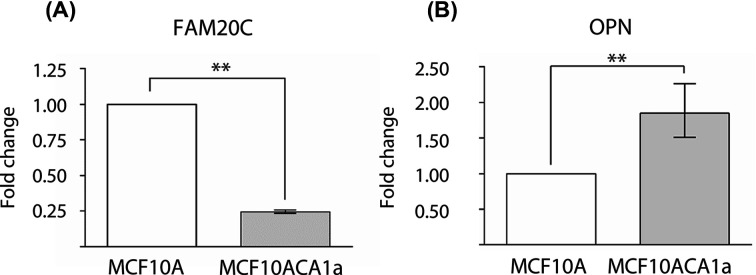
FAM20C and OPN expression in MCF10A and malignant MCF10ACA1a cells (**A**) FAM20C mRNA level and (**B**) OPN mRNA level from MCF10A and MCF10ACA1a cells. Relative FAM20C and OPN mRNA levels were quantified by qRT-PCR analysis. FAM20C and OPN levels from normal MCF10A cells were set as reference and expressions from MCF10ACA1a are presented as mean ± S.D. (*n*=3). ** indicates *P*<0.0001. The data are representative of two independent experiments.

To investigate whether FAM20C mRNA expression was also reduced in another *ras*-transformed cell line, expression levels were compared between MCF10A cells and its malignant H-*ras* transformed and PIK3CA H1047R mutated counterpart, MCF10ACA1a. The comparison showed that the mRNA expression of FAM20C in malignant MCF10ACA1a cells was reduced four-fold compared with that of the MCF10A cells (Figure 3A). The mRNA expression of OPN was also analyzed and found to be approximately two-fold higher relative to that in MCF10A cells (Figure 3B). Only faintly stained OPN fragments migrating below 25 kDa, and no intact OPN, were detected in the medium conditioned by the MCF10A and MCF10ACA1a cells (Supplementary Figure S1).

### FAM20C-transfection of *ras*-transformed 275-3-2 cells

**Figure 4:**
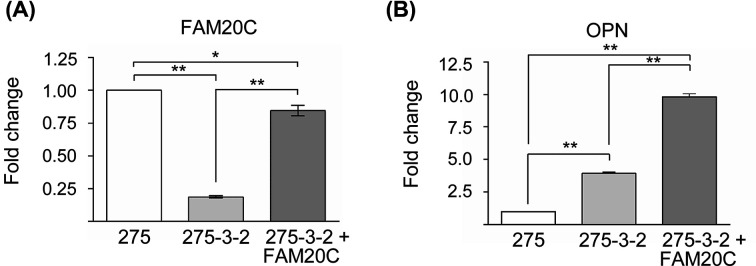
FAM20C and OPN mRNA expression after transfection with FAM20C (**A**) FAM20C and (**B**) OPN mRNA levels in *ras*-transformed 275-3-2 cells before and after FAM20C-transfection were quantified by qRT-PCR. The FAM20C and OPN levels in 275 cells are set as reference. The results are shown as mean ± S.D. (*n*=3). * and ** indicate *P*<0.01 and *P*<0.0001, respectively. The experiments were repeated three times.

The *ras-*transformed 275-3-2 cells were transfected with an FAM20C-encoding plasmid, which resulted in an increase in FAM20C expression from 18 to 85% of the level in non-transformed 275 cells (Figure 4A). The OPN mRNA expression was increased ten-fold in the FAM20C transfected *ras*-transformed 275 cells compared with the 275 cells (Figure 4B).

**Figure 5:**
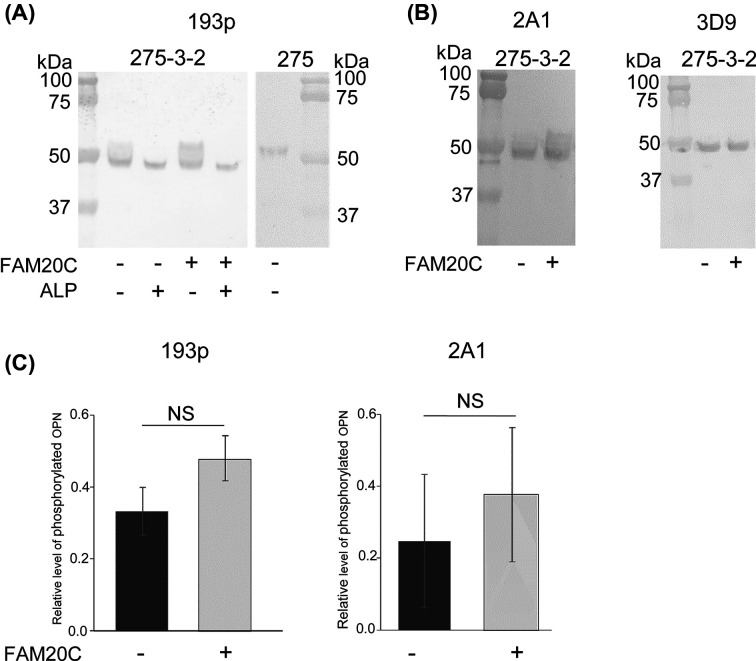
Phosphorylation of OPN in *ras*-transformed 275-3-2 cells after transfection with FAM20C Conditioned medium from the cells was analysed using SDS/PAGE on a 10% bis-tris gel and Western blotting was performed with the monoclonal OPN antibodies 193p, 2A1 and 3D9 (**A,B**). Dephosphorylation with ALP is indicated. (**C**) Quantification of the OPN band intensities detected by the 193p and 2A1 antibodies. The ratio between the intensity of phosphorylated OPN (upper band) and the intensity of OPN in both bands is shown as mean ± S.D for three independent Western blots.

Next, it was analyzed whether the increased FAM20C mRNA expression in these cells resulted in an increased phosphorylation of fOPN^ras^. Conditioned medium from 275, *ras*-transformed 275-3-2 and FAM20C-transfected *ras*-transformed 275-3-2 cells was analyzed by Western blotting. Western blotting using the 193p antibody of the conditioned medium of *ras*-transformed 275-3-2 cells showed a strong OPN band migrating just below the 50 kDa marker and a fainter band migrating just above 50 kDa (Figure 5A). The bands represent two OPN phosphorylation isoforms, where ALP treatment showed that the higher molecular form contains more phosphorylations than the lower molecular weight form (Figure 5A). Furthermore, the 2A1 antibody, but not the 3D9 antibody, detected the indistinct band above 50 kDa, confirming the phosphorylation of the upper band (Figure 5B). After FAM20C-transfection, the intensity of the upper band detected by 193p and 2A1 representing the more phosphorylated OPN isoform appeared to be higher, but the increase was not significant (Figure 5C).

## Discussion

In the present study, we show that OPN from *ras*-transformed fibroblasts is less phosphorylated than OPN from the non-transformed parental cells (Figure 1). It has been shown that *ras*-transformation and cancerogenity are associated with OPN isoforms that are less phosphorylated compared with OPN from a non-transformed source [12,24,34]. Furthermore, less phosphorylated OPN and highly phosphorylated OPN have been observed to co-exist in tumor tissue [16]. It has been suggested that the less phosphorylated OPN and highly phosphorylated OPN are secreted from cancer cells and macrophages, respectively [16,22,23]. Different OPN phospho-isoforms have been associated with functional differences as, e.g. less phosphorylated OPN from *ras*-transformed fibroblasts has been shown to promote significantly stronger cell adhesion compared with highly phosphorylated OPN from osteoblasts [12]. The present study compares the phosphorylation level of OPN from *ras*-transformed fibroblasts and the parental non-transformed cells, and thereby directly analyzes the differences in OPN phosphorylation after *ras*-transformation.

The mRNA expression of OPN was increased four-fold in *ras*-transformed 275-3-2 cells compared with 275 cells, which correlated with higher OPN protein secretion by the *ras*-transformed cells (Figure 2A,B). This confirms the well-established observation that OPN mRNA expression is increased in *ras*-transformed and cancer cells [35]. It could be hypothesized that the increased OPN mRNA expression results in OPN protein levels exceeding what can be correctly phosphorylated by FAM20C. Another explanation for the reduced phosphorylation of OPN could be a decrease in the expression of FAM20C in the transformed cells. The observation that the mRNA expression of FAM20C was reduced in *ras*-transformed 275-3-2 cells to only 18% of the level observed in the non-transformed 275 cells supports this hypothesis (Figure 2C). Attempts of comparing FAM20C protein levels between the to cell lines using immunoblotting with specific FAM20C antibodies were unsuccessful (Supplementary Figure S2). This problem has also been reported by others as the amount of endogenous FAM20C kinase is very low [36].

In other studies using cells not influenced by *ras*-transformation, transfection with FAM20C resulted in increased phosphorylation levels of several FAM20C substrates including OPN [1,2]. To examine whether increased FAM20C expression in the *ras*-transformed cells would promote higher OPN phosphorylation levels, the *ras*-transformed 275-3-2 cells were transfected with FAM20C. This increased the FAM20C mRNA levels from 10 to 85% of the levels in the non-transformed 275 cells (Figure 4A). Based on Western blotting, the restored mRNA levels of FAM20C did not not translate into a significant increase in phosphorylation of OPN in the *ras*-transformed 275-3-2 cells (Figure 5). A possible explanation for this could be the ten-fold increase in the endogenous OPN mRNA levels after FAM20C-transfection of the *ras*-transformed 275-3-2 cells compared with the 275 cells (Figure 4B). The reason for this dramatic increase in OPN expression after FAM20C transfection is not known. The inefficiency of the FAM20C kinase to increase OPN phosphorylation in FAM20C-transfected *ras*-transformed 275-3-2 cells could also be due to undiscovered mechanisms in the *ras*-transformed phenotype. Sphingosine and the pseudokinase family with sequence similarity, member A (FAM20A) have been identified as activators of FAM20C kinase activity [36,37]. A decreased expression or inhibition of these activators could therefore potentially contribute to a decreased activity of FAM20C.

Comparison of the human mammary epithelial cell line, MCF10A, and its malignant counterpart, MCF10ACA1a, showed that the mRNA expression of FAM20C in MCF10ACA1a was reduced to 24% of the level in MCF10A. Furthermore, the OPN mRNA expression was increased approximately two-fold (Figure 3). However, the expressed OPN from both these cell lines could not be analysed by Western blotting, as only low molecular weight OPN fragments and not the intact OPN protein were detected (Supplementary Figure S1). The reduction in FAM20C mRNA expression and the increase in OPN mRNA expression after *ras*-transformation in both murine fibroblasts cells and in human mammary cells suggests that this could be a general phenomenon of *ras*-transformed cells. Interestingly, the two cell lines, *ras*-transformed 275-3-2 and malignant MCF10ACA1a, were both transformed with *H-ras*. The cancer phenotype of MCF10ACA1a was further promoted due to a subsequent activating *PIK3CA H1047R* mutation [31]. However, since *H-ras* occured as a proto-oncogene in both cell lines, it could be hypothesized that the FAM20C promoter contains a *ras*-responsive element that consequently inhibits FAM20C transcription.

FAM20C is responsible for the phosphorylation of the majority of secreted phosphoproteins and impaired activity of FAM20C have been associated with many pathological conditions [4–8]. Hence, reduced expression of FAM20C observed in relation to *ras*-transformation can likewise be hypothesized to affect the phosphorylation and functions of a plethora of secreted proteins.

In summary, we have shown that OPN is less phosphorylated in *ras*-transformed 275-3-2 cells compared with the parental 275 cell line. We have shown that FAM20C mRNA expression is significantly decreased after *ras*-transformation in the two cell lines, 275-3-2 and MCF10ACA1a. This could indicate that part of the oncogenic phenotype generated by *ras*-transformation includes a reduction in the expression of the FAM20C kinase.

## Supplementary Material

Supplementary Figure S1-S3

## Abbreviations

ALP: alkaline phosphatase
DMEM: Dulbecco’s modified Eagle’s medium
FAM20C: family with sequence similarity 20, member C
fOPN: OPN from murine embryo fibroblast 275 cell
fOPN^ras^: OPN from the ras-transformed 275-3-2 cell
OPN: osteopontin
qRT-PCR: quantitative reverse transcription PCR
