# Supplementary material for: Ras-transformation reduce FAM20C expression and osteopontin phosphorylation
Source: Biosci Rep. 2020 Sep 16;40(9):BSR20194378. doi: 10.1042/BSR20194378 (PMC7494989; doi:10.1042/BSR20194378)

Supplementary Figure S1

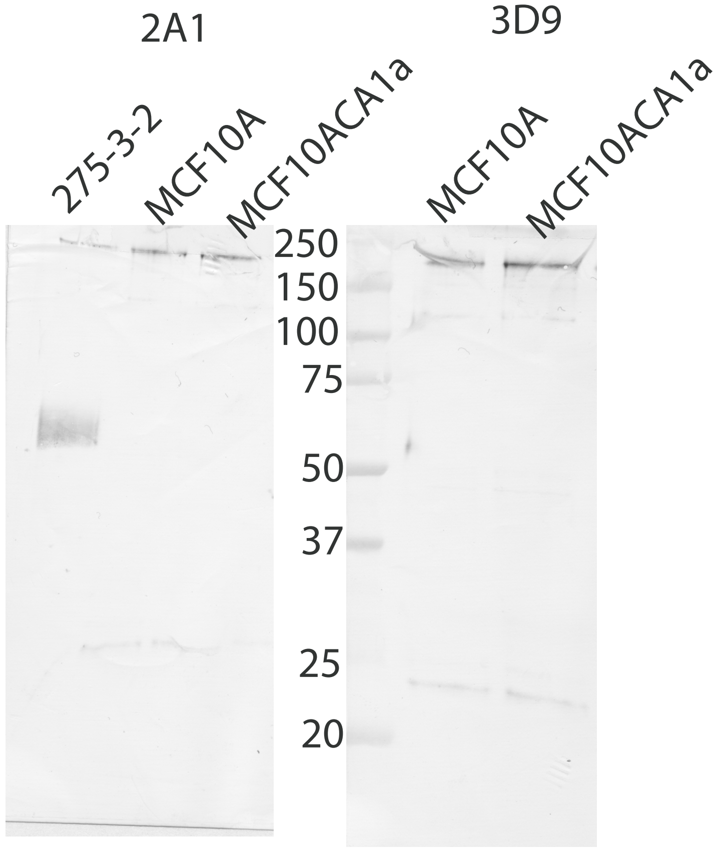

Figure S1. Western blotting of OPN secreted by MCF10A and malignant MCF10ACA1a cells. OPN from conditioned medium was separated on a 16% tris-tricine gel by SDS-PAGE followed by western blotting using the monoclonal OPN antibodies 2A1 and 3D9.

Supplementary Figure S2

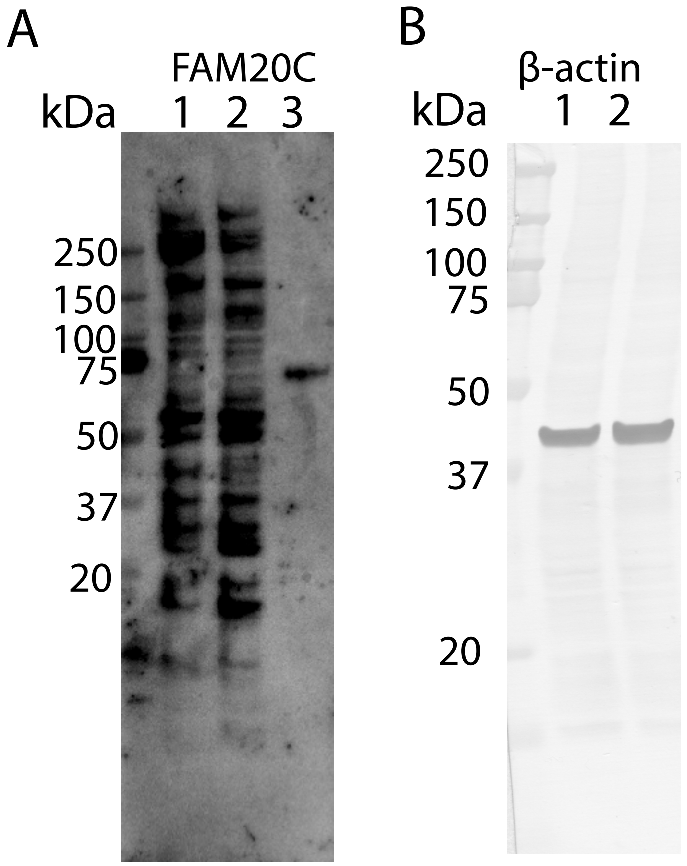

Figure S2. Western blot analysis of FAM20C in 275 and 275-3-2 cells. Cell lysates from 275 and *ras*-transformed 275-3-2 cells were analysed by western blotting with a polyclonal FAM20C antibody (A) or a monoclonal β-actin antibody (B). Lane 1, 275 cell lysate; lane 2, 275-3-2 cell lysate and lane 3, Fam20C standard.

Supplementary Figure S3

Uncropped western blots

From Figure 1:

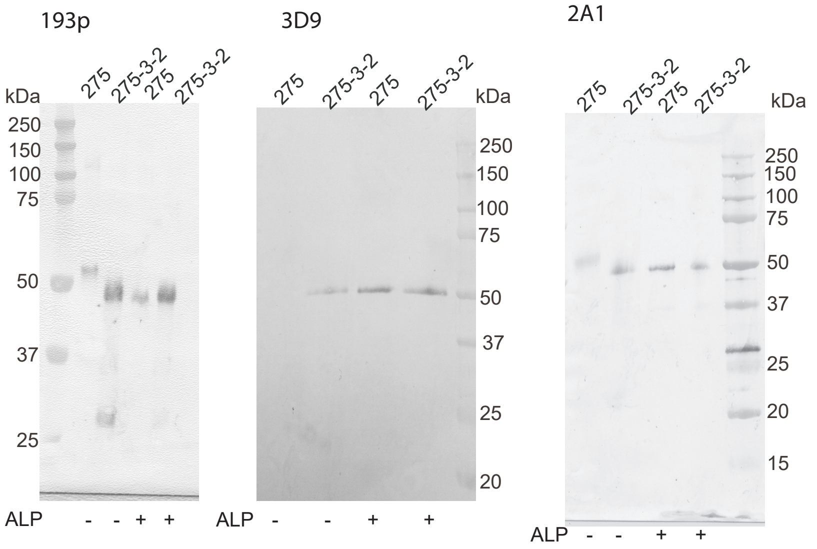

From Figure 5:

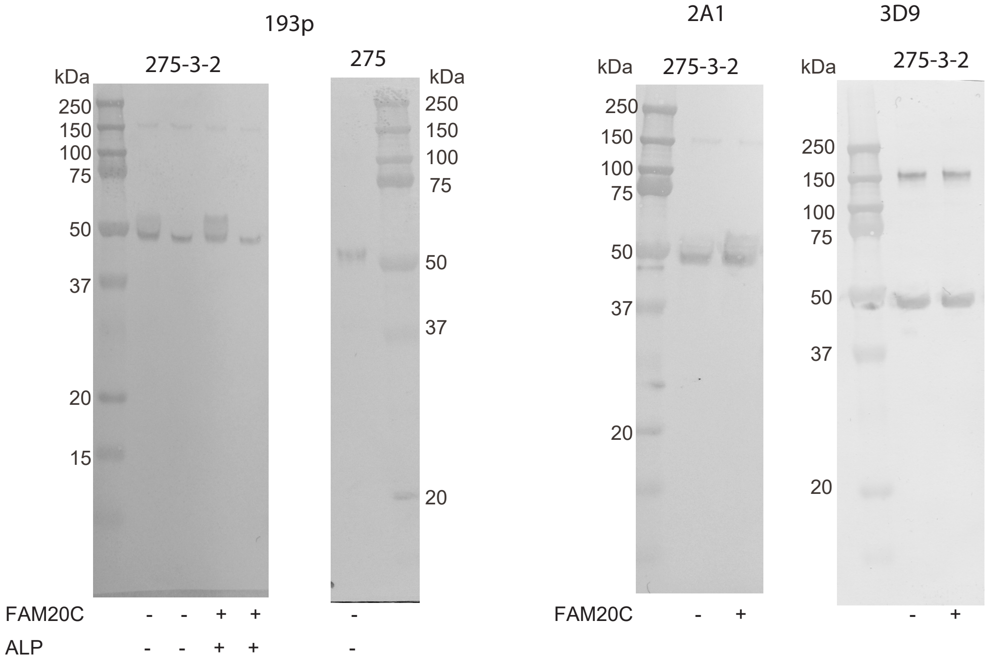

Supplement: Supplementary Figure S1-S3 [file BSR-2019-4378_supp.pdf]
